# Physical Activity Assessment Between Consumer- and Research-Grade Accelerometers: A Comparative Study in Free-Living Conditions

**DOI:** 10.2196/mhealth.6281

**Published:** 2016-09-19

**Authors:** Gregory M Dominick, Kyle N Winfree, Ryan T Pohlig, Mia A Papas

**Affiliations:** ^1^ University of Delaware Department of Behavioral Health and Nutrition Newark, DE United States; ^2^ Informatics and Computing Program Northern Arizona University Flagstaff, AZ United States; ^3^ University of Delaware Biostatistics Core Facility Newark, DE United States

**Keywords:** Fitbit, activity tracker, actigraphy, physical activity, aerobic exercise, validity

## Abstract

**Background:**

Wearable activity monitors such as Fitbit enable users to track various attributes of their physical activity (PA) over time and have the potential to be used in research to promote and measure PA behavior. However, the measurement accuracy of Fitbit in absolute free-living conditions is largely unknown.

**Objective:**

To examine the measurement congruence between Fitbit Flex and ActiGraph GT3X for quantifying steps, metabolic equivalent tasks (METs), and proportion of time in sedentary activity and light-, moderate-, and vigorous-intensity PA in healthy adults in free-living conditions.

**Methods:**

A convenience sample of 19 participants (4 men and 15 women), aged 18-37 years, concurrently wore the Fitbit Flex (wrist) and ActiGraph GT3X (waist) for 1- or 2-week observation periods (n=3 and n=16, respectively) that included self-reported bouts of daily exercise. Data were examined for daily activity, averaged over 14 days and for minutes of reported exercise. Average day-level data included steps, METs, and proportion of time in different intensity levels. Minute-level data included steps, METs, and mean intensity score (0 = sedentary, 3 = vigorous) for overall reported exercise bouts (N=120) and by exercise type (walking, n=16; run or sports, n=44; cardio machine, n=20).

**Results:**

Measures of steps were similar between devices for average day- and minute-level observations (all *P* values > .05). Fitbit significantly overestimated METs for average daily activity, for overall minutes of reported exercise bouts, and for walking and run or sports exercises (mean difference 0.70, 1.80, 3.16, and 2.00 METs, respectively; all *P* values < .001). For average daily activity, Fitbit significantly underestimated the proportion of time in sedentary and light intensity by 20% and 34%, respectively, and overestimated time by 3% in both moderate and vigorous intensity (all *P* values < .001). Mean intensity scores were not different for overall minutes of exercise or for run or sports and cardio-machine exercises (all *P* values > .05).

**Conclusions:**

Fitbit Flex provides accurate measures of steps for daily activity and minutes of reported exercise, regardless of exercise type. Although the proportion of time in different intensity levels varied between devices, examining the mean intensity score for minute-level bouts across different exercise types enabled interdevice comparisons that revealed similar measures of exercise intensity. Fitbit Flex is shown to have measurement limitations that may affect its potential utility and validity for measuring PA attributes in free-living conditions.

## Introduction

Accelerometers have been extensively used in research to objectively measure and quantify changes in physical activity (PA) and sedentary behavior [1,2]. However, research-grade accelerometers are prohibitively expensive [3] and require extensive training for data collection and analysis [4]. Low-cost wearable activity monitors, such as Fitbit, have become widely available to consumers, enabling users to self-monitor and track their daily PA levels, steps, energy expenditure (EE), and distance as well as diet and sleep patterns over time [5,6]. Many of these devices include user interface features through a mobile phone app that also provide behavior change strategies such as self-monitoring, goal setting, feedback provision, and social support communicated via push notifications, email, and social media platforms (eg, Facebook) [7]. Given that 21% of US adults report using technology to track personal health data [6], there is considerable potential for using these commercial devices in research settings as they appeal to consumers and researchers alike owing to their relatively low-cost, user-friendly apps, and potential to improve health [8-10].

Fitbit is one of the most popular brands of consumer-grade, wearable, activity tracking monitors, accounting for more than 50% of over 3 million devices sold worldwide between 2013 and 2014 [11]. Several Fitbit models can be worn at the hip (Fitbit Ultra, Zip, One) and, more recently, the wrist (Fitbit Flex, Charge, and Surge). A recent systematic review summarized the findings of 22 studies published between 2012 and 2015 that examined the validity and reliability of different wearable activity monitors for measuring steps, EE, and, to a lesser degree, moderate-to-vigorous physical activity (MVPA) [12]. Overall, 20 studies reported on at least one type of Fitbit device (Ultra, Zip, One, Flex) and findings generally indicate that Fitbit may be a valid instrument for measuring steps compared with direct observation and objective accelerometer assessment (eg, ActiGraph GT3X); however, greater measurement error has been reported during slower walking speeds [12-14]. Researchers have also examined the extent to which Fitbit provides accurate EE estimates against criterion measures such as indirect calorimetry [15-18] and accelerometry [4]. In general, these studies indicate that Fitbit underestimates EE across different modes of activity [4,16,18] and overestimates EE when activities are combined [15]. It has been suggested that the variability in EE estimates observed for individual types of activity (eg, sedentary, aerobic, and resistance exercises) offsets the overall EE estimates calculated by Fitbit [15]. To date, most Fitbit validation studies are limited to the measurement of steps and EE using waist-worn Fitbit devices with the majority of this research being conducted in controlled laboratory settings with short observation periods [12]. Extending this research to examine the variation of activity as it occurs over time in free-living conditions can improve current knowledge about the measurement properties of the wrist-worn Fitbit Flex device.

Few studies have examined the accuracy of Fitbit compared with ActiGraph for measuring MVPA in free-living conditions [4,19,20]. One study reported that step counts and minutes of MVPA measured over 7 days were strongly correlated between the Fitbit Zip and ActiGraph GT3X; however, interdevice differences for quantifying MVPA were not reported [19]. Ferguson and colleagues [4] found that the Fitbit One and Fitbit Zip were each correlated with ActiGraph GT3X+ for minutes of MVPA (*r*=.91 and .88, respectively) measured over a 48-hour period; however, both Fitbit devices overestimated time in MVPA by as much as 137 minutes and 157 minutes, respectively. In addition, the study relied on a consensus approach to approximate MVPA cut points, and a short observation period limits the generalizability of these findings [4]. Considering this limited evidence, additional research is needed to examine the relative agreement between Fitbit and ActiGraph for measuring steps and time in different intensity levels over longer periods of time in free-living conditions.

By using the Fitbit application programming interface provided through a third-party service provider (eg, Fitabase, Small Steps Labs LLC), it is now possible to obtain data for steps, PA intensity, EE, as well as metabolic equivalent task (MET) estimates, stratified by specific time intervals (eg, hour- and minute-level data), that were previously unavailable to researchers. Because METs reflect oxygen consumption rates in relation to intensity, these values can be used to confirm proprietary intensity thresholds used by Fitbit to indicate a person’s activity level [21]. Yet it is unclear how METs derived from Fitbit compare with METs determined by ActiGraph. Furthermore, by accessing Fitbit data across different intervals of time it is possible to examine how measurement differences vary in free-living conditions.

The purpose of this study was to examine the concordance between the Fitbit Flex and ActiGraph GT3X accelerometer for measuring steps, METs, and proportion of time spent in sedentary behavior and in light, moderate, and vigorous PA in a sample of young adults in free-living conditions. A second study aim was to compare steps, METs, and intensity between ActiGraph and Fitbit across minutes of reported exercise and by exercise type.

## Methods

### Participants

A convenience sample of 19 young adult men (n=4) and women (n=15) who owned a Fitbit Flex device volunteered to participate in this study. Participants were considered apparently healthy and were recruited from within the University of Delaware. Approval from the university’s institutional review board was obtained before the study. Data were collected between October and November 2014.

### Instruments and Measures

#### Fitbit Flex

The Fitbit Flex (Fitbit Inc, San Francisco, CA) is a small, wireless device that fits within a wristband and uses a triaxial accelerometer to convert raw acceleration signals into counts. These counts are then applied to proprietary algorithms that provide estimates of steps/minute, PA level (sedentary, light, moderate, vigorous), and EE [22-24]. Because raw acceleration data are not stored on the Fitbit device, researchers must rely on the converted activity counts determined by Fitbit. A Fitabase account was created for this study in order to obtain daily and minute-level Fitbit data that included estimated METs.

#### ActiGraph GT3X

The ActiGraph GT3X (ActiGraph, Pensacola, FL) is a research-grade triaxial accelerometer that is approximately the size of a standard pedometer and is typically worn at the waist to provide objective measures of sedentary and PA behavior in free-living conditions [3]. The proprietary ActiLife software allows researchers to choose from several validated algorithms to quantify PA depending on the participant pool (eg, toddlers or preschoolers, adults, and older adults). The extent to which the ActiGraph GT3X quantifies data is contingent on the specific cut point and scoring algorithms that a researcher selects. Generally, PA level (sedentary, light, moderate, vigorous, and very vigorous), step count, EE, and METs are computed from the accelerometer counts detected within a specified time period (ie, epoch). Data can be calculated for different time intervals (week, day, hour, and minute-level).

### Procedures

Data collection procedures were staggered over a 2-month period. Consenting participants provided their Fitbit Flex username and password that linked their device to the Fitabase platform for continuous data collection throughout the study period. At baseline, participants completed a standard demographics questionnaire that included age, sex, and race or ethnicity (African American, Asian or Pacific Islander, Caucasian, Hispanic or Latino, other). Next, anthropometric data were obtained by a trained technician using standard procedures. Standing height and weight were obtained with participants in bare feet and light clothes via stadiometer (Seca, Chino, CA) and digital scale (Seca, Chino, CA), measured to the nearest 0.1 cm and 0.1 kg, respectively. Body mass index (BMI) score was calculated as the weight in kilograms divided by height in meters squared (kg∙m^−2^). Percent body fat was determined from bioelectrical impedance (Bodystat Ltd, Isle of Man, UK). After completing the baseline measures, participant data (birth date, sex, height, weight) were used to initialize Fitbit Flex and ActiGraph GT3X devices. Participants were then instructed to simultaneously wear the Fitbit Flex and ActiGraph GT3X on their dominant side (wrist and hip, respectively) during all waking hours for 7 consecutive days. An exercise journal was provided in which participants were instructed to list up to 4 daily bouts of “purposeful exercise” including the type of exercise and start and end times of each bout performed. Participants were also asked to report if they did no purposeful exercise on any of the 7 days.

After 7 days, participants returned to the laboratory to confirm wear time compliance, and their completed exercise journals were collected. All participants were asked to complete a second 7-day wear period that for most (n=16) occurred approximately 3 weeks after the first wear period. Data collection procedures for the second wear period remained the same, except that demographic and anthropometric data were not assessed a second time. Participants received a US $15 gift card for each 7-day measurement period they completed.

### Data Processing

ActiGraph GT3X data were processed using the ActiLife software version 6.11.9. Wear times were validated using the Troiano (2007) algorithm [25]. Nonwear periods were defined if no epoch counts were detected over a period of ≥60 continuous minutes. All participants wore both devices for a minimum of 8 waking hours over 7 consecutive days. Estimated METs and PA cut points were determined using the validated Freedson adult vector magnitude algorithm (2011) [26]. ActiGraph data were aggregated to 60-second epochs and the vigorous and very vigorous PA categories were combined to be consistent with Fitbit Flex PA data.

Minute-level Fitbit Flex data were downloaded from the Fitabase server. Data were registered based on time so that minute-level measures from Fitbit and ActiGraph were consistent. We considered Fitbit nonwear periods if a 0 was recorded for ≥60 continuous minutes. Data were excluded if either ActiGraph or Fitbit indicated periods of nonwear. Self-reported exercise bouts were considered valid if the days and minutes of reported exercise matched to within 5 minutes of the activity counts that were concurrently measured with ActiGraph. Because Fitbit provides overall estimates of EE (eg, total calories) and ActiGraph provides EE estimates from PA only, a decision was made to exclude EE in this study.

### Measurement

Outcome measures included steps, METs, and proportion of time in sedentary activity and light, moderate, and vigorous PA determined by ActiGraph and Fitbit. Data were examined at two levels: (1) daily average of 14 days and (2) minutes of all reported exercise and by exercise type. Similar to previous studies [27,28], we defined sedentary behavior using a cut point threshold of < 200 counts/minute with an additional criterion that no steps were recorded. To compare intensity levels between devices for minutes of reported exercise and exercise type, an average intensity score was created by applying the numerical code used by Fitbit to define intensity levels for each minute of reported exercise (0 = sedentary, 1 = light, 2 = moderate, and 3 = vigorous) to the same minute-level vector magnitude counts from ActiGraph: sedentary (0 = 0-199 counts), light (1 = 200-2690 counts), moderate (2 = 2691-6166 counts), and vigorous (3 = counts ≥ 6167).

Reported exercise bouts (N=120) were noted and exercise types were grouped into 6 categories: walk, run or sports, bike, cardio dance, cardio machine, and weights. Because of the wide variation in reported types of exercises, a decision was made to provide results for the 3 most homogeneous of those categories: (1) run or sports included dynamic aerobic activities such as running, jogging, basketball, football, soccer, and ultimate Frisbee; (2) cardio machine included stationary, machine-based aerobic exercises such as walking, jogging, or running on a treadmill and using the elliptical trainer, stair-climber, and stationary bike; and (3) walking exclusively outdoors.

### Statistical Analyses

Participants’ characteristics are reported using means and standard deviations for continuous variables and frequencies and percentages for categorical variables. All PA metrics were treated as continuous variables and are reported with means and standard deviations. Analyses were conducted using two different models: first, using simple paired samples *t* tests and second, using generalized linear mixed modeling (GLMM). The GLMM accomplishes the same comparisons as the paired samples *t* test but allows observations to be nested within individuals. Given that the intraclass correlations for the GLMM were small (< .25) and results for both models were equivalent, the simpler method’s findings are presented. Furthermore, as recommended by the American Statistical Association [29], we wanted to draw particular attention to the effect sizes, rather than just relying on *P* values to demonstrate the magnitude of measurement differences between the devices; currently, GLMM does not provide a way to garner effect sizes. Relative agreement between devices is presented using correlations. Alpha for the study was set at the nominal level, alpha = .05; statistical significance was determined using *P* value < .05. Given the relatively small size of the study sample, effect sizes (Cohen’s *d*) are also reported. Data were analyzed using SPSS version 23 (IBM Corp, Armonk, NY, USA).

## Results

### Participants

Participant characteristics (N=19) and PA levels, averaged over 14 measurement days, are reported in Table 1. Overall, most participants were female (15/19, 79%). Participants’ ages ranged between 19 and 37 years. Ranges for BMI and percent body fat values for male and female participants were 18.5-28.0 kg∙m^−2^ and 11.4%-37.1%, respectively. Overall, 74% (14/19) of the participants were white (male, n=2; female, n=12); approximately 11% (2/19) of the participants were Latino (male, n=1; female, n=1). Remaining racial or ethnic groups included Asian or Pacific Islander, black, and other; n=1 in each group (data not shown). On average, participants spent the most time in sedentary activity followed by light-intensity PA (67.8%, SD 6.6%, and 25.2%, SD 5.8%, respectively) and less than 10% of the time in MVPA. Average time in MVPA per day ranged between 25 and 137 minutes. Overall, participants reported 153 hours of exercise (6588 total minutes) reflecting 94 person-days, of which 120 unique bouts of exercise were identified. Participants reported an average of 6.34 exercise bouts over 14 days (median = 5.5 bouts, range = 1-22 bouts; data not shown).

**Table 1:** Participant characteristics (N=19) and physical activity levels (determined from ActiGraph GT3X) averaged over 14 days.

Variables	Male (n=4)		Female (n=15)	
	Mean (SD)	Range	Mean (SD)	Range
Age, years	20.0 (0.8)	19.0-21.0	21.3 (4.4)	19.0-37.0
Height, cm	174.4 (6.6)	165.7-181.6	162.2 (7.9)	145.8-173.1
Weight, kg	77.2 (5.1)	72.0-83.9	57.4 (10.9)	39.3-80.2
Body mass index, kg.m^2^	24.6 (1.0)	23.2-25.5	21.9 (2.7)	18.5-28.0
Body fat, %	13.5 (1.5)	11.4-14.7	25.9 (5.7)	15.4-37.1
Sedentary, %	62.1 (4.7)	57.0-70.0	69.5 (6.2)	58.0-82.0
Light intensity, %	28.5 (2.4)	25.0-33.0	24.2 (6.1)	15.0-36.0
Moderate intensity, %	8.5 (3.1)	5.0-13.4	5.1 (1.7)	2.59-8.27
Vigorous intensity, %	0.9 (0.9)	0.0-2.55	1.1 (1.8)	0.0-6.77
MVPA^a^, %	9.4 (2.9)	4.9-13.9	6.2 (2.9)	2.7-14.5
Average MVPA, minutes/day	83.4 (25.2)	40.7-120.3	55.4 (28.3)	25.0-137.7

^a^MVPA: moderate-to-vigorous physical activity.

### Measurement Differences for Average Day-Level Activity

Average daily MET rate, proportion of time in sedentary activity and light, moderate, and vigorous PA, and total steps/day are reported in Table 2. interdevice correlations indicated good agreement for total steps/day (*r*=.91); moderate agreement for time in vigorous PA (*r*=.80); low agreement for daily MET rate (*r*=.70), time in sedentary activity (*r*=.67), and time in light PA (*r*=.68); and very low agreement for time in moderate PA (*r*=.43). The devices did not significantly differ for estimates of total steps/day (*P*=.10, *d*=0.40). Compared with ActiGraph, the Fitbit Flex significantly overestimated daily MET rate (mean difference 0.7, SD 0.09, METs/day, *P*<.001, *d*=3.76), proportion of time in sedentary activity (mean difference 26.0% per day, *P*<.001, *d*=4.33), proportion of time in moderate PA (mean difference 3.0%, SD 11.0%, per day, *P*<.001, *d*=0.86), and proportion of time in vigorous PA (mean difference 3.0%, SD 1.0%, per day, *P*<.001, *d*=2.21). Fitbit significantly underestimated the proportion of time in light PA compared with ActiGraph (mean difference −34.0%, SD −3.0%, per day, *P*<.001, *d*=6.90).

**Table 2:** Average day-level differences for activity variables between devices (N=19).

Variable	Device	Mean (SD)	*r* ^a^	Cohen’s *d*^b^	*P* value
MET^c^ rate/day			.70	3.76	< .001
	ActiGraph	1.3 (0.17)			
	Fitbit	2.0 (0.26)			
Sedentary activity (daily %)			.67	4.33	< .001
	ActiGraph	43.0 (7.0)			
	Fitbit	69.0 (7.0)			
Light intensity (daily %)			.68	6.90	< .001
	ActiGraph	49.0 (7.0)			
	Fitbit	15.0 (4.0)			
Moderate intensity (daily %)			.43	0.86	< .001
	ActiGraph	7.0 (3.0)			
	Fitbit	10.0 (14.0)			
Vigorous intensity (daily %)			.80	2.21	< .001
	ActiGraph	2.0 (2.0)			
	Fitbit	5.0 (3.0)			
Total step count/day			.91	0.40	.10
	ActiGraph	9639.41 (3456.47)			
	Fitbit	10,286.08 (3760.31)			

^a^Pearson correlation.

^b^Cohen effect size.

^c^MET: metabolic equivalent task.

### Minute-Level Measurement Differences for Reported Exercise (N=120)

Table 3 reports the average minute-level step count, MET rate, and intensity score for 120 bouts of reported exercise (6588 minutes). Correlations indicate the devices had moderate agreement for steps/minute (*r*=.85) and low agreement for MET rate/minute (*r*=.70) and intensity score/minute (*r*=.65). The devices did not significantly differ for estimates of total steps/minute (*P*=.559, *d*=0.04) or intensity score/minute (*P*=.057, *d*=0.04). The Fitbit Flex significantly overestimated MET rate/minute compared with ActiGraph (mean difference 1.8, SD 0.42, METs/minute, *P*<.001, *d*=0.18).

**Table 3:** Measurement differences for overall minutes of reported exercise bouts by device (N=120).

Variable	Device	Mean (SD)	*r*	Cohen’s *d*	*P*
MET rate/minute			.70	0.18	< .001
	ActiGraph	4.12 (2.93)			
	Fitbit	5.92 (3.35)			
Intensity score/minute			.65	0.04	.057
	ActiGraph	1.83 (0.77)			
	Fitbit	1.96 (0.92)			
Step count/minute			.85	0.04	.559
	ActiGraph	77.60 (51.64)			
	Fitbit	76.10 (51.17)			

^a^Pearson correlation.

^b^Effect size.

^c^MET: metabolic equivalent task.

^d^Mean intensity score (range: sedentary = 0, light = 1, moderate = 2, vigorous = 3).

### Minute-Level Measurement Differences by Exercise Type

Results were similar when minute-level measures between Fitbit and ActiGraph were examined by exercise type (Table 4). There were 16 bouts of walking ranging from 20 to 136 minutes (mean 68.0, SD 37.0, minutes), 44 bouts of run or sports ranging from 15 to 130 minutes (mean 52.0, SD 30.0, minutes), and 20 bouts of cardio-machine exercise ranging from 31 to 66 minutes (mean 47.0, SD 10.0, minutes).

For minutes of walking-based exercise, Fitbit significantly overestimated MET rate (mean difference 3.16, SD 1.54, METs/minute, *P*<.001, *d*=1.34) and mean intensity score (mean difference 0.51, SD 0.25, *P*=.007, *d*=0.78) compared with ActiGraph. No significant measurement differences were found for walking steps/minute (*P*>.05). For minutes of run or sports exercise, no interdevice differences were found for mean intensity score or for steps/minute (*P*>.05 for both). However, Fitbit significantly overestimated MET rate compared with ActiGraph (mean difference 2.0, SD 0.72, METs/minute, *P*<.001, *d*=0.78). There were no significant differences between ActiGraph and Fitbit for estimated MET rate, mean intensity score, or step count for minutes of cardio-machine exercise (all *P* values >.05).

**Table 4:** Minute-level measurement differences for activity variables by exercise type and device.

Exercise type	Variable	ActiGraph mean (SD)	Fitbit mean (SD)	*r* ^a^	Cohen *d*^b^	*P* value
Walking (n=16)						
	MET^c^ rate/minute	2.37 (1.38)	5.53 (2.92)	.60	1.34	< .001
	Intensity/minute^d^	1.47 (0.60)	1.98 (0.85)	.64	0.78	.007
	Step count/minute	62.93 (48.25)	70.29 (45.89)	.80	0.23	.346
run or sports (n=44)						
	MET rate/minute	5.61 (2.83)	7.61 (3.55)	.70	0.78	< .001
	Intensity/minute	2.22 (0.66)	2.31 (0.83)	.66	0.16	.308
	Step count/minute	106.37 (49.00)	103.48 (51.77)	.73	0.08	.377
Cardio machine (n=20)						
	MET rate/minute	3.37 (0.76)	3.04 (0.68)	.54	0.38	.108
	Intensity/minute	2.19 (0.92)	2.30 (0.98)	.57	0.12	.588
	Step count/minute	99.86 (50.67)	94.37 (47.43)	.52	0.04	.619

^a^Pearson correlation.

^b^Cohen effect size.

^c^MET: metabolic equivalent task.

^d^Mean intensity score range: sedentary = 0, light = 1, moderate = 2, vigorous = 3.

## Discussion

### Principal Findings

Fitbit Flex and ActiGraph GT3X provided consistently similar step counts for average daily activity, overall minutes of reported exercise (N=120), and minutes of reported walking, run or sports, and cardio machine types of exercises. Mean intensity scores were generally comparable for overall minutes of reported exercise and for run or sports and cardio-machine exercise types. However, significant measurement differences were found for the average daily proportion of time in sedentary activity and light-, moderate-, and vigorous-intensity PA. Significant differences for MET rate estimates were found between Fitbit and ActiGraph for average daily activity, overall minutes of reported exercise, and also differed between devices for average day activity, overall minutes of reported exercise, and minutes of reported walking and run or sports exercises.

### Average Daily and Minute-Level Step Counts

This study found that Fitbit Flex was strongly correlated with ActiGraph GT3X for steps measured per day and for overall minutes of reported exercise (*r*=.91 and .85, respectively), which is consistent with previous research using hip-worn versions of Fitbit (eg, Zip and One) [4,19] and, more recently, Fitbit Flex [13,20,30]. Although step counts were not significantly different between ActiGraph and Fitbit for day- and minute-level observations, interdevice agreement varied when steps/minute were examined by exercise type (walking, *r*=.80; run or sports, *r*=.73; cardio machine, *r*=.52). This finding is similar to Bai and colleagues [15] who reported that EE error estimates for ActiGraph and Fitbit Flex were approximately 17% when measured bouts of sedentary activity as well as aerobic and resistance exercises were combined. However, when examined separately, error estimates for all monitors increased and varied by activity type. The authors suggested that the lower error estimates observed for the combined protocol was likely due to an overall cancelation of inaccurate measurement estimates, which became evident once the activities were examined individually [15]. Although EE was not examined in our study, the relationship between step counts, MET rate, intensity, and EE is strongly supported by the literature [31,32]. Given that Fitbit uses step-count data to partially inform the algorithms used to estimate minute-level METs, intensity, and EE [22,23], it is likely that METs and time in different intensity levels will also vary depending on the unit of analyses (eg, overall daily activity vs minutes of exercise) and by exercise type. It is also possible that the variation in steps observed between Fitbit Flex and ActiGraph are due to differences in device placement (wrist vs hip). Previous studies have shown that the placement of activity monitors may be more or less sensitive to different body positions, movement patterns, and speeds [30,33]. Compared with researcher-counted steps, wrist- and hip-worn Fitbit devices are shown to underestimate step estimates during stationary cycling, whereas the Fitbit Flex may be more likely to underestimate steps during walking activities [30]. Our findings extend this earlier work by quantifying steps over a longer observation period in absolute free-living conditions that included analyses for day-level activities and minutes of self-reported exercise.

### Daily and Minute-Level Metabolic Equivalent Tasks

Significant measurement differences were found between Fitbit and ActiGraph for estimating METs for average daily activity as well as overall minutes of reported exercise bouts. Although the mean difference in average daily METs was relatively small (0.7, SD 0.09), differences became larger for minute-level analyses (1.8, SD 0.42, METs), despite having moderately strong correlations across observation periods (*r*=.70 for both). It is probable that the small distribution in MET values contributed to this finding.

When minute-level exercise categories were examined, MET values were not significantly different between devices for cardio-machine exercise only. The finding that Fitbit overestimated METs for walking (+3.16 METs) and run or sports (+2.0 METs) exercises suggests that the algorithm Fitbit uses to estimate METs differs from the selected algorithm that was applied to ActiGraph data. For example, the mean MET estimates for walking were 5.53 (SD 2.92) and 2.37 (SD 1.38) for Fitbit and ActiGraph, respectively. From these results, walking METs derived from Fitbit approach vigorous intensity (ie, ≥ 6.0 METs), whereas walking METs estimated by ActiGraph indicate less than moderate intensity (ie, 3.0 METs) [21]. Differences in device placement may have also contributed to these findings. Although no studies are known to have measured differences in MET estimates between Fitbit and ActiGraph, previous research has reported that Fitbit Flex overestimates EE compared with ActiGraph during aerobic exercise [15]. Alternatively, a participant could have misreported his or her exercise information, for example, listing a single bout of exercise in which both walking and jogging activities were performed but were specifically recorded as “walking” or “jogging” could cause the data to be misclassified in the analyses. This explanation may also provide insight into why MET values were not different for cardio-machine exercises, as these “stationary” exercises (eg, elliptical) may be more easily recalled by participants and less likely to result in misclassification; however, further research is needed to verify this assertion. It is also possible that both devices are not sensitive enough to detect stationary-based activity, although MET estimates from Fitbit Flex and ActiGraph appear to be appropriate for moderate-intensity exercise. Future studies are needed to examine whether alternative placement of Fitbit devices improves measurement accuracy during stationary exercise, as step count and EE estimates are shown to be more accurate when research-grade accelerometers are placed on the ankle or thigh [34,35].

### Average Daily Proportion of Time in Different Intensity Levels

The average proportion of time spent in sedentary activity and light, moderate, and vigorous PA per day was calculated at the day-level only. Results indicate that Fitbit and ActiGraph provided significantly different measures in all intensity levels. It is challenging to make interdevice comparisons of intensity level because interpretation of results is constrained by differences in how accelerometer counts from Fitbit and ActiGraph are used to measure intensity. A major barrier to studies examining the measurement validity and reliability of Fitbit is the proprietary laws that prevent researchers from understanding how Fitbit determines the cut points used to classify different intensity levels, although it is reasonable to believe that the cut points used by Fitbit are not consistent with those used in research with ActiGraph. It is well documented that using different accelerometer cut points produces different MVPA outcomes [36]. This “cut-point nonequivalence” prevents comparisons across studies that use different cut points [37]. It appears that this issue now extends to commercial activity tracking devices. Efforts to incorporate signal features and patterns from raw acceleration data may help develop more sophisticated models to improve activity intensity estimates [1,38].

### Mean Intensity Scores for Overall Minutes of Exercise and Exercise Category

Mean intensity scores were calculated for every minute of all reported exercise bouts (N=120) and by specific exercise category (walking, run or sports, and cardio machine). When reported minutes of exercise bouts were examined, overall mean intensity scores were marginally similar between Fitbit (1.96, SD 0.92) and ActiGraph (1.83, SD 0.77), *P*=.057. However, scores became notably more equivalent when exercises were grouped by category, except for walking (Fitbit = 1.98, SD 0.85; ActiGraph = 1.47, SD 0.60; *P*=.007). Although the small sample size of the walking category (n=16) likely influenced this finding, it is reasonable that combining the wide distribution of different walking-based activities and variation in walking speeds may have also led to these differences given that Fitbit is shown to overestimate intensity at slower walking speeds and underestimate intensity at faster walking speeds compared with direct measures of EE [13]. Future research is needed to examine the precise cut points used by Fitbit to define light, moderate, and vigorous intensity, particularly with walking-based exercise.

To our knowledge, this is the first study to examine the congruence of PA intensity estimates between Fitbit and ActiGraph using a mean intensity score. Given that the proportion of time in various intensity levels was different when device-specific cut points were examined, this approach enabled comparisons to be made between ActiGraph and Fitbit Flex that revealed that overall intensity was not significantly different for exercises performed at presumably higher intensity levels (eg, run or sports and cardio machine). Other approaches to compare intensity level between Fitbit Flex and ActiGraph have also been reported. Alharbi and colleagues [20] used METs obtained from Fitbit to approximate minutes of MVPA in a sample of cardiac rehabilitation patients (N=48) who wore the Fitbit Flex and ActiGraph GT3X over 4 days. Although the Fitbit Flex was found to overestimate MVPA by 10 minutes/day compared with ActiGraph GT3X, the device was found to be highly accurate for identifying patients who met the minimum PA guidelines of 150 minutes of MVPA per week [20]. However, results from our study suggest that Fitbit overestimates METs and therefore may not accurately reflect time in MVPA. Clearly, additional work is needed to identify methods that reduce the cut-point nonequivalence between Fitbit and ActiGraph accelerometers.

### Methodological Considerations

The results of this study add to the existing literature and advance current knowledge related to PA metrics as measured by Fitbit and ActiGraph in truly free-living conditions. Assessments included observational wear periods of up to 14 days—longer than any published study to date [12]. Analyses examined measurement differences for daily average and minute-level observation periods that included 120 individual exercise bouts that were also categorized by type: walking, running/sports, and cardio machine. We also provide a detailed account as to how the devices were initialized and data were collected as recommended [12]. Moreover, we examined differences in METs and the proportion of time in various intensity levels, which is also limited in the literature [12,20]. Despite these strengths, the use of a small, convenience sample that included healthy, nonoverweight, and highly active young adults limits the generalizability of these results. Although the addition of 94 person-days of reported exercise enhanced the robustness of data collected, future research should include a larger and more diverse population. Whereas data collected from both devices were time synchronized, differences in device placement (wrist vs waist) were likely susceptible to differences in upper and lower body movement [34,35]. Although others have used similar device placements [4,20], future studies are needed to substantiate these findings using wrist-worn, research-grade accelerometers. This study also relied on self-reported exercise that is inherently subject to recall and response bias [39]. However, self-report data provide context that can complement accelerometer-based data collected in free-living conditions [1].

At the time this study was implemented (2014), the Fitbit Flex was the most recent device available and the existing literature was primarily limited to a small number of laboratory-based studies that examined the validity of earlier, waist-worn Fitbit devices (eg, Zip and One) [12]. Since then, Fitbit has released 6 different models (Charge, Charge HR, Surge, Alta, and Blaze) that offer new and more advanced features including heart rate monitoring and geographic information systems. On the basis of current literature, it is clear that the rate at which this technology is currently developed and marketed to consumers greatly outpaces the rigor of scientific investigation, and proprietary laws prevent transparency regarding the potential utility of these devices in research. Additionally, because software changes can be made at any time, this can greatly influence the measurement properties of the device and can negate findings that have been previously reported [12]. In 2015, Fitbit changed its algorithm for defining MVPA to align more closely with the Centers for Disease Control and Prevention’s recommendations [40], in which Fitbit reports active minutes only when activity levels of ≥3 METs are recorded for 10 or more continuous minutes [23]. Because we examined minute-level intensity scores (obtained through Fitabase) and matched these to minutes of reported exercise, it is unlikely that these changes affected the outcomes of this study. However, researchers should use caution when relying on data directly obtained from a user’s Fitbit dashboard.

### Conclusions

The results of this study indicate that the Fitbit Flex provides reasonably accurate estimates for steps and overall mean intensity scores for which exercise bouts are reported, particularly for activities other than walking. However, the algorithm that Fitbit uses to estimate MET rate is not equivalent to ActiGraph in the generalized case. This study also highlights the measurement disparity between day-level and minute-level observations, as well as measurement differences for specific types of exercises. However, the lack of transparency regarding the measurement properties used by Fitbit and routine changes that are made to the Fitbit software and firmware can affect the way Fitbit measures different PA attributes. This perpetually calls into question the validity and reliability of Fitbit data and has implications regarding the applicability of Fitbit in research settings. Future research by our investigative team will include modeling the intensity levels determined by research-grade accelerometers to those used by Fitbit in order to create a more standardized method of measurement and improve the feasibility of using Fitbit in applied PA research.

## Abbreviations

BMI: body mass index
EE: energy expenditure
GLMM: general linear mixed modeling
METs: metabolic equivalent tasks
MVPA: moderate-to-vigorous physical activity
PA: physical activity
